# Lethal autonomous weapons systems, revulsion, and respect

**DOI:** 10.3389/fdata.2022.991459

**Published:** 2022-10-20

**Authors:** Richard Dean

**Affiliations:** ^1^United States Naval Academy, James B. Stockdale Center for Ethical Leadership, Annapolis, MD, United States; ^2^Department of Philosophy, California State University, Los Angeles, Los Angeles, CA, United States

**Keywords:** lethal autonomous weapons systems (LAWS), artificial intelligence, military ethics, respect, conventional attitudes, revulsion

## Abstract

The potential for the use of artificial intelligence in developing lethal autonomous weapons systems (LAWS) has received a good deal of attention from ethicists. Lines of argument in favor of and against developing and deploying LAWS have already become hardened. In this paper, I examine one strategy for skirting these familiar positions, namely to base an anti-LAWS argument not on claims that LAWS inevitably fail to respect human dignity, but on a different kind of respect, namely respect for public opinion and conventional attitudes (which Robert Sparrow claims are strongly anti-LAWS). My conclusion is that this sort of respect for conventional attitudes does provide some reason for actions and policies, but that it is actually a fairly weak form of respect, that is often override by more direct concerns about respect for humanity or dignity. By doing this, I explain the intuitive force of the claim that one should not disregard public attitudes, but also justify assigning a relatively weak role when other kinds of respect are involved.

## Introduction

Robert Sparrow has argued that since there is widespread moral revulsion at the idea of developing and deploying Lethal Autonomous Weapons Systems (LAWS), to do so would show disrespect for humans who have this feeling or attitude of revulsion (Sparrow, 2016). Given some (controversial) empirical assumptions, I think he may be right that respecting this conventional attitude provides a *prima facie* reason to reject LAWS. But keeping in mind a distinction between this kind of symbolic respect and a deeper and more foundational form of respect for persons, it can be seen that Sparrow's proposed respect for conventional attitudes only provides a fairly weak, *prima facie* reason for action. This is important, because it acknowledges the strong aversion that some people have to the possibility of developing LAWs, but simultaneously suggests limits on the argumentative role of this feeling.

### Hypothesis

It is possible to accommodate the important moral intuition that one ought to show a kind of respect for widespread attitudes and conventions, without supposing that such attitudes provide an overriding reason to reject all use of LAWS.

### Method

I will examine existing views on the role of respect for conventional attitudes on the moral permissibility of developing and employing LAWS, extract the key elements of these views, and argue that these views can be accommodated without a comprehensive ban on LAWS.

## Method: Moral debate about LAWS

The longstanding trend toward using military weapons from great distances has accelerated in recent decades, with the use of artillery and aerial bombardment being supplemented by precision guided missiles and remotely piloted aircraft. In addition to the increasing distance between combatants and their targets, some weapons systems have the capability to operate in ways that are significantly autonomous, or independent of direct human control, the movement toward increasingly remote operation of lethal weapons and the movement toward systems capable of operating without direct human control have not fully intersected. Weapons capable of operating fully autonomously have either been defensive, like the US Aegis missile defense system (when on “auto special” setting), or they have been directed at targets other than humans, like Israel's Harop system, which is designed to destroy radar equipment. However, this historical separation of the autonomy of weapons systems and their lethal application to human targets apparently has collapsed recently, as the first uses of autonomous drones against human targets, without human oversight, reportedly have already occurred (Cramer, 2021).^1^

The anticipatory moral debate about developing and deploying such LAWS has been lively and passionate. It is widely agreed that the technological limits of current AI make it ethically unjustified to assign decisions about targeting humans to weapons systems, independent of human oversight—that is, to employ weapons systems against human targets with human controllers “out of the loop,” to use current terminology. While AI may well distinguish between allied and enemy forces in many battlefield conditions, it is not yet as reliable as humans in making more subtle judgments about whether enemy combatants are *hors de combat*, or at identifying irregular forces, making judgments about whether civilians are actively supporting military operations, or foretelling collateral damage and civilian casualties. But it seems inevitable that the AI employed in LAWS will eventually become at least as good as humans at tasks like this. The question of whether there is a principled reason to prohibit the use of LAWS at that point is a matter of heated debate, and a number of lines of moral argument for and against their use have become firmly entrenched.

The most fundamental principled objection to the development and use of LAWS is that removing human control from the process of targeting and killing human beings would in some way show disrespect for humankind, or would be a failure to recognize and acknowledge human dignity (Asaro, 2012). The objection turns on a claim that respect for human dignity requires some kind of active recognition of the humanity of the target, a recognition of which machines are inherently incapable.^2^ But a response to this objection also has become standard, namely that by this standard, LAWS should be no less acceptable than many weapons that have been in use for many years or even decades, such as cruise missiles or standard artillery (Jenkins and Purvis, 2016).

Proponents of the use of LAWS have developed their own influential argument, emphasizing that when LAWS become better than human operators at distinguishing between legitimate military targets and civilians, the lives saved will constitute such a positive consequence that the use of LAWS will not be morally wrong, but may instead be morally required (Arkin, 2010). This pro-LAWS argument also can be presented as a matter of respecting the dignity of the humans whose lives are saved, directly countering the argument that use of LAWS must be eschewed for the sake of respecting human dignity (Jenkins and Purvis, 2016).

The basic positions have been staked out for several years, with a good deal of the moral thinking on the topic of LAWS consisting more or less of modifications and reinforcements within this framework (Skerker et al., 2020; Bohrer, 2022; Kahn, 2022). In this kind of hardened rhetorical landscape, it is useful to try new approaches, and that seems to be the motivation for an argument offered by Robert Sparrow, in which respect and disrespect play a different role than in the standard anti-LAWS position.

## Results: Sparrow's “conventional respect” argument against LAWS

Sparrow relies on an idea that what counts as respectful or disrespectful can depend on “social understandings” so there is a “conventional element to our understanding of the requirements of respect” (Sparrow, 2016, p. 109). Sparrow's approach may circumvent the need to settle some of the standard, highly controversial questions about human dignity, respect, and their role in debates about LAWS. This is because instead of attempting to establish theoretically that the nature of human dignity and respect for persons requires a direct and personal engagement with any person who is affected by life and death decisions, it substitutes a different kind of respect, namely respect for conventional attitudes. To flout these widespread attitudes sends a message of disrespect to people who have them, by implying that their feelings or attitudes are unimportant.

Sparrow's strategy here places great weight on a supposed “widespread public revulsion at the idea of autonomous weapons” (Sparrow, 2016, p. 109), and that “Most people already feel strongly that sending a robot to kill would express a profound disrespect of the value of an individual human life” (Sparrow, 2016, p. 109). Sparrow admits that “it is possible that public revulsion at sending robots to kill people will be eroded as AWS come into use and become a familiar feature of war” (Sparrow, 2016, p. 116), but he regards this as a significant change from what he takes to be the currently prevalent attitude, that the use of LAWS would be an appalling example of failure to respect human dignity. Sparrow says, “the strength and popular currency of the intuition that the use of [LAWS] would profoundly disrespect the humanity of those they are tasked to kill is sufficient justification to try to establish such a prohibition” (Sparrow, 2016, p. 111).

Although I will grant, for the sake of argument, Sparrow's premise that there is widespread public revulsion at the thought of the use of LAWS, this is only a hypothetical concession, and in fact the premise is not strongly supported. Sparrow's main evidence is a 2013 survey, in which 39% of respondents said they “strongly oppose” the use of “robotic weapons that can independently make targeting and firing decisions without humans in the loop,” and 16% said they “somewhat oppose” it (Carpenter, 2013). However, the survey did not ask why respondents held their views, so it is hasty to conclude that even the 55% of respondents who opposed the use of LAWS did so because of a feeling of repugnance. There are many other reasons why someone might oppose the use of LAWS against humans, including concerns about the technical adequacy of AI in targeting, a general pacifism, or a resistance to increasing the gap between nations with advanced military technology and those without. A 2021 survey by Human Rights Watch is more suggestive of some public unease or perhaps revulsion at the use of LAWS, though it is still inconclusive. Asked whether they support or oppose the use of LAWS (with the concept of LAWS being explained within the survey question), 41.9% of all respondents said they “strongly oppose” LAWS and 19.4% “somewhat oppose” it. This survey also asked respondents who opposed the use of LAWs for the reasons for their opposition. It found that 66.2% of all respondents who oppose the use of LAWS said, “They'd cross a moral line because machines should not be allowed to kill” (Human Rights Watch, 2021). There is room for concern about circularity—use of LAWS is wrong because it crosses a line into wrongness—but the idea of a “moral line” also is at least compatible with a feeling of moral revulsion. So, charitably, it may be that about 40% of all respondents (two-thirds of the 63% of respondents who opposed the use of LAWS) feel at least some unease or perhaps even revulsion at the thought of military use of LAWS. This is some potential evidence, but weak evidence, for the claim that there is a widespread feeling of revulsion toward LAWS.

Nevertheless, it is worth granting this contestable claim, to see how strong an argument against the use of LAWS can be formulated, using it as a premise. Not only does the 2021 survey mentioned above hint at some revulsion, but it also is undoubtedly the case that some commentators on LAWS display strong and deeply held feelings about the repugnance of allowing machines to make decisions about lethally targeting humans, and about the incompatibility of this practice with human dignity. Besides the authors Sparrow mentions (e.g., Gubrud, 2014), many other examples could be cited (e.g., Heyns, 2017). It is unclear what amount of public revulsion is needed in order to count as supporting a moral requirement of respecting the attitude. The answer to this question is neither obvious nor empirically resolvable, but would instead itself be a matter for moral argument. Instead of embarking on that project, I will grant hypothetically that there is a “strong” feeling of revulsion at the thought of using LAWS, and see what argument follows.

Sparrow does not simply take a feeling of moral revulsion to provide direct proof that some practice, like the use of LAWS, is wrong. That strategy, which is sometimes (probably uncharitably) attributed to Kass (1997), seems implausible.

Instead, Sparrow's argument works through an idea of societal conventions about what counts as respectful or disrespectful treatment. Although Sparrow does not go into great detail about the connection between feelings of revulsion and conventions regarding respect, his thought appears to be that feelings of revulsion about some practice are one indication that deeply held conventions or attitudes regarding respect are being violated. This is consistent with his example of a deep feeling of disgust in reaction to the mutilation of corpses, even though what counts as mutilation “is conventional and may change over time” (Sparrow, 2016, p. 109). Even though a specific way of treating corpses (cutting fingers off, eating parts of them, burning them) may be regarded as disrespectful according to the conventional standards of one society or a set of societies, the conventions of some other society might deem the same treatment respectful. But these ways of treating corpses genuinely are disrespectful in virtue of violating conventions, despite the conventions being mutable, because violating conventions about respect is a way to exhibit disrespect. If some society has a convention against touching strangers with one's left hand, then deliberately touching a stranger with one's left hand in this society is disrespectful, whether the origins of the convention have to do with health and hygiene, religion, combat practices, or just superstition, and regardless of whether some other society holds any such convention. In the same way, Sparrow maintains that attitudes of revulsion at the thought of employing LAWS show that there is a widely shared conventional attitude that assigning decisions about taking a life to machines is disrespectful to human dignity, and that violating these conventions is a way of showing disrespect. “That the boundaries of such respect are sometimes—as in this case—determined by convention (in the sense of shared social understandings rather than formal rules) does not detract from the fact that it is fundamental to the ethics of war” (Sparrow, 2016, p. 110).

So, a simple representation of Sparrow's argument would be:

(a) If some type of action violates conventions of respect and disrespect, then this is a moral reason not to perform this type of action.
(b) The development and use of LAWS violates widespread conventions regarding respect and disrespect for humans, which require personal recognition and acknowledgment of the life being taken (and revulsion at the idea of using LAWS is evidence of this violation of conventions).
(c) Therefore, we have a moral reason not to develop and deploys LAWS.

This representation of Sparrow's argument is deliberately vague (in that it leaves open how compelling a reason is provided by respect for conventions), and examination reveals that if more properly specified, it is a sound argument, but that it also is limited in the ramifications of its (fairly weak) conclusion.

## Discussion: Sparrow's argument and two levels of respect

There is something intuitively compelling about Sparrow's argument. It does seem morally problematic to flout conventions regarding respectful and disrespectful behavior, or to dismiss feelings of revulsion at possibly grave violations of some of these conventions. But the question is how much weight to give to these norms and attitudes, compared to other considerations involved in the possible use of LAWS.

In a paper directly responding to Sparrow's position, Purves and Jenkins acknowledge that “public aversion to a technology counts against its adoption,” but they are quick to dismiss some of these attitudes, because the attitudes are not based on sound moral reasons (Purves and Jenkins, 2016, p. 396). They say that “public opinion can be swayed by an array of factors, only some of which are indicative of the moral truth of a matter,” and that “ethicists should not be satisfied to let public opinion carry the day, especially in the absence of a robust moral distinction…” (Jenkins and Purvis, 2016, p. 396). In effect, they question premise 1 of the reconstruction of Sparrow's argument offered above—they claim that conventions of respect should only be accommodated if they are based on sound moral reasoning. Some thought experiments can be generated in support of this position. For example, suppose that some nation at war pleads with enemy forces to only use combatants of northern European descent in military engagements with them. “Please do not allow people of color to take our lives,” they say, “This is deeply disrespectful of our traditions, which maintain that only people of our own race are worthy opponents.” Intuitively, it seems that such a plea should carry no weight, because it is based on misguided, racist, moral ideals.

But, despite cases like this, it is hasty to conclude that we should respect only feelings and conventions that we think are based on sound moral reasons. Suppose that instead of pleading that no person of color should be deployed as a combatant against them, the nation at war pleads that ammunition used against them should contain no copper. “Our spiritual and religious convictions tell us that copper is impure, and contaminates our souls,” they say. If ammunition were available that did not contain copper, and if its use were as effective as ammunition containing copper, then it seems that their revulsion at the idea of being killed by copper should carry moral weight, providing at least some moral reason to use non-copper ammunition. And this would be so, even if their aversion to copper seemed to be based on mere superstition, instead of any sound moral reasoning. For that matter, the example of avoiding touching strangers with one's left hand seems to be a real way of showing respect, even if the origins of the convention are disconnected from any current negative or positive effects.

Instead of saying that conventions of respect should carry weight only if they can be seen to rest on sound moral reasoning, a more nuanced account is needed. Our own choices, especially if they are deliberate policy decisions, not only lead to actions, but also send a message. In this sense, Sparrow is correct that “ethics is a realm of meanings” (Sparrow, 2016, p. 101). I can deliberately flout conventions of respect and send a message that those conventions are morally objectionable, or I can simply fail to consider them, and send a message that I disrespect those who hold the conventional attitudes. Or I can adhere to the conventions of respect even if I do not see their point, sending a message of respect for those who do regard the conventions as important. This picture, encompassing the communicative element of actions, allows for a subtler, more nuanced picture of the role of conventions. However, it is of little pragmatic help in telling us how much weight to give to some particular, controversial conventions or attitudes, such as the attitude that the use of LAWS is contrary to human dignity.

But a point about respect drawn from normative moral theory is useful here, and suggests that in the case of LAWS, respect for conventions or attitudes provides a relatively weak reason for action, which may well be outweighed by other considerations. This can be seen by disambiguating the concept of “respect,” which is used in many different ways in moral discourse.

Sparrow's argument centers on a specific type of respectful action, namely actions that have to do with adherence to or rejection of conventions. Such conventions can encompass matters like not touching strangers with one's left hand, or more profound matters, such as how corpses are handled or how lethal combat decisions are made. We can show respect by adhering to conventions, respect both for individuals affected by our actions and respect for those who hold the conventions. But there must be a rationale for recognizing and adhering to any class of duties, including these duties of conventional respect.

And this deeper rationale for recognizing various classes of duties is sometimes also described by using the word “respect,” in a different sense. Kantian theories often make respect for persons, or for the dignity of humanity, the deep basis of many or all duties. So Wood takes respect for the dignity of rational nature as the basis of all duties to oneself or others (Wood, 2008). Darwall, whose views have a looser affinity with Kant's, identifies a type of respect he calls “recognition respect,” and takes it that recognition respect for persons is the basis of our duties regarding what one person can morally demand of another (Darwall, 1977). Hill takes from Kant a moral requirement to treat all persons with “basic respect” as potential moral decision-makers, with this basic respect leading to more specific moral requirements (Hill, 2000). The strategy of grounding moral claims on a foundational respect for persons or for dignity is not restricted to moral theory, it is also displayed in important public statements, such as the United Nations' Universal Declaration of Human Rights, which grounds its specific human rights on “recognition of the inherent dignity” of all humans, and requires universal and equal “respect” for these rights (United Nations, 1948).

This foundational role that respect for persons or for human dignity often plays imbues the word “respect” with an aura of moral significance, even inviolability. After all, if all duties are based on respect for persons, then to fail to give this kind of respect is by definition wrong. Even if only some substantial subset of duties are based on respect for persons, then it would take powerful countervailing reasons to override these duties.

But a derivative set of duties are more likely to be defeasible, even if the duties go by the name of duties of “respect.” Symbolic respect for persons, instantiated in actions such as trying to abide by conventions of respect, may be based on a foundation of deep respect for persons, but may be frequently overridden by other duties that also are based on foundational respect for persons. In fact, it appears that duties such as preventing loss of life, distributing goods and outcomes fairly, and maybe even being truthful, often outweigh symbolic respect for conventions, in a moral system based on a foundational respect for persons. There is *prima facie* reason to abide by a convention against touching strangers with one's left hand, but this reason becomes inert if the only way to save a stranger from a burning car is to pull her out with both hands. There is some moral reason to eschew ammunition containing copper in order to show symbolic respect for a society's conventional attitudes, but if the war can be ended more quickly and with much less suffering by using ammunition containing copper, the prohibition on copper falls away easily. And, in a military context, even the convention of adhering strictly to rank hierarchies, which is quite strong in modern militaries, can be outweighed when one is given orders that violate the standard rules of *jus in bello*, which can plausibly be seen as being based on respect for humanity.

While I do not claim that requirements of respect for conventions are so weak as to always be outweighed by any conflicting moral considerations, it does seem that they are often relatively weak *prima facie* duties, if one keeps in mind the distinction between duties of respect for specific conventions and the deep foundational respect that many ethicists take to be central to morality. If so, then Sparrow has identified a source of the intuition that conventional attitudes of repugnance toward the use of LAWS provide some reason to refrain from developing and using LAWS. But it is a weak reason, that does not obviously seem to outweigh other morally important factors, such as minimizing loss of life or self-defense against unjust attacks.

## Data availability statement

The original contributions presented in the study are included in the article/supplementary material, further inquiries can be directed to the corresponding author.

## Author contributions

The author confirms being the sole contributor of this work and has approved it for publication.

## Funding

This study was supported by PRIO, Peace Research Institute Oslo.

## Conflict of interest

The author declares that the research was conducted in the absence of any commercial or financial relationships that could be construed as a potential conflict of interest.

## Publisher's note

All claims expressed in this article are solely those of the authors and do not necessarily represent those of their affiliated organizations, or those of the publisher, the editors and the reviewers. Any product that may be evaluated in this article, or claim that may be made by its manufacturer, is not guaranteed or endorsed by the publisher.

## Footnotes

^1^Although even simple weapons, such as land mines or IEDs, can operate without direct human control, I am following the parameters of the debate started by Sparrow and others, in focusing on lethal weapons systems directed at human targets that involve some discrimination or targeting with humans out of the loop. (Some “automatic” weapons like the Russian POM-3 mines may blur the line between automatic and more fully autonomous weapons, in that they do discriminate between human and other moving targets).

^2^As an anonymous reviewer has noted, the response below presupposes that one can view a machine as distinct from the intention of its creator. So, besides the standard response I describe, a more radical response is possible, in which machines as artifacts can express a variety of attitudes of their human creators.

## References

[B1] Arkin R. (2010). The case for ethical autonomy in unmanned systems. J. Military Ethics 9, 332–341. 10.1080/15027570.2010.536402

[B2] Asaro P. (2012). On banning autonomous weapon systems: human rights, automation, and the dehumanization of lethal decision-making. Int. Rev. Red Cross 94, 687–709. 10.1017/S1816383112000768

[B3] Bohrer A. (2022). The Moral Case for the Development of Autonomous Weapons Systems. Available online at: https://blog.apaonline.org/2022/02/28/the-moral-case-for-the-development-of-autonomous-weapon-systems/ (accessed June 20, 2022).

[B4] Carpenter C. (2013). US Public Opinion on Autonomous Weapons. Available online at: http://www.duckofminerva.com/wp-content/uploads/2013/06/UMass-Survey_Public-Opinion-on-Autonomous-Weapons.pdf (accessed June 20, 2022).

[B5] Cramer M. (2021, June 3). AI drone may have acted on its own in attacking fighters, U.N. says. New York Times.

[B6] Darwall S. (1977). Two kinds of respect. Ethics 88, 36–49. 10.1086/292054

[B7] Gubrud M. (2014). Stopping killer robots. Bull. Atomic Sci. 70, 32–42. 10.1177/0096340213516745

[B8] Heyns C. (2017). Autonomous weapons in armed conflict and the right to a dignified life: an African perspective. South Afr. J. Hum. Rights 33, 46–71. 10.1080/02587203.2017.1303903

[B9] Hill T. (2000). Respect, Pluralism, and Justice: Kantian Perspectives, Oxford: Oxford University Press.

[B10] Human Rights Watch (2021). Autonomous Weapons. Available online at: https://www.ipsos.com/sites/default/files/ct/news/documents/2021-01/ipsos_global_advisor_-_lethal_autonomous_weapons_survey_-_nov_2020-jan_2021.pdf (accessed June 20, 2022).

[B11] Jenkins R. Purvis D. (2016). Robots and respect: a response to Robert Sparrow. Ethics Int. Affairs 30, 391–400. 10.1017/S0892679416000277

[B12] Kahn L. (2022). Lethal autonomous weapons systems, jus in bello, and respect for human dignity. Cybersecurity and Privacy, a section of the journal Frontiers in Big Data. 10.3389/fdata.2022.999293 PMC9500208 36156937

[B13] Kass L. (1997). The wisdom of repugnance. New Republic 32, 17–26. 11654974

[B14] Skerker M. Purves D. Jenkins R. (2020). Autonomous weapons systems and the moral equality of combatants. Ethics Inform. Technol. 22, 197–209. 10.1007/s10676-020-09528-0

[B15] Sparrow R. (2016). Robots and respect: assessing the case against autonomous weapons systems. Ethics Int. Affairs. 30, 93–116. 10.1017/S0892679415000647

[B16] United Nations (1948). Universal Declaration of Human Rights. Available online at: https://www.un.org/en/about-us/universal-declaration-of-human-rights (accessed June 20, 2022).

[B17] Wood A. (2008). Kantian Ethics. Cambridge: Cambridge University Press.

